# Sunitinib versus Pazopanib Dilemma in Renal Cell Carcinoma: New Insights into the In Vitro Metabolic Impact, Efficacy, and Safety

**DOI:** 10.3390/ijms23179898

**Published:** 2022-08-31

**Authors:** Filipa Amaro, Carolina Pisoeiro, Maria João Valente, Maria de Lourdes Bastos, Paula Guedes de Pinho, Márcia Carvalho, Joana Pinto

**Affiliations:** 1 Associate Laboratory i4HB, Department of Biological Sciences, Laboratory of Toxicology, Faculty of Pharmacy, University of Porto, 4050-313 Porto, Portugal; 2 UCIBIO-REQUIMTE, Department of Biological Sciences, Laboratory of Toxicology, Faculty of Pharmacy, University of Porto, 4050-313 Porto, Portugal; 3 National Food Institute, Technical University of Denmark, Kongens Lyngby, 2800 Copenhagen, Denmark; 4 FP-I3ID, FP-BHS, University Fernando Pessoa, 4200-150 Porto, Portugal; 5 Faculty of Health Sciences, University Fernando Pessoa, 4200-150 Porto, Portugal

**Keywords:** metastatic renal cell carcinoma, tyrosine kinase inhibitors, toxicity, oxidative stress, metabolomics, ^1^H NMR spectroscopy, endometabolome, exometabolome

## Abstract

Sunitinib and pazopanib are tyrosine kinase inhibitors (TKIs) used as first-line therapy for metastatic renal cell carcinoma (RCC). Although these TKIs are associated with similar survival outcomes, some differences have been reported in their safety profiles. In this work, traditional toxicological endpoints (cell viability and growth, oxidative stress, and nuclear morphology) and ^1^H NMR spectroscopy-based metabolomics analysis were used to provide new insights into the cytotoxicity and metabolic mechanisms underlying sunitinib and pazopanib treatments. Tumoral (Caki-1) and non-tumoral (HK-2) human renal cells were exposed to clinically relevant concentrations of sunitinib (2 µM) or pazopanib (50 µM). Sunitinib showed selectivity for cancer cells, inhibiting proliferation, and inducing apoptotic death of Caki-1 cells, whereas pazopanib had a similar cytotoxic effect in both tumoral and non-tumoral cells. ^1^H-NMR metabolomics unveiled a higher impact of sunitinib on the levels of intracellular metabolites of Caki-1 cells (seven dysregulated metabolites), suggesting dysregulations on amino acid, glutathione and glycerophospholipid metabolisms. In contrast, pazopanib had a higher impact on the levels of extracellular metabolites of Caki-1 cells (seven dysregulated metabolites in culture medium), unveiling alterations on amino acid and energetic metabolisms. In HK-2 cells, sunitinib caused only a minor increase in intracellular isoleucine levels, whereas pazopanib induced several alterations on the intracellular (three dysregulated metabolites) and extracellular (three dysregulated metabolites) compartments suggesting changes on amino acid, glycerophospholipid, and energy metabolisms. Our results demonstrate that these TKIs elicit distinct cellular and metabolic responses, with sunitinib showing better in vitro efficacy against target RCC cells and lesser nephrotoxic potential than pazopanib.

## 1. Introduction

Renal cell carcinoma (RCC) is the most common form of kidney cancer and is responsible for about 2.2% of all cancer diagnoses and 1.8% of deaths worldwide [1,2]. The clear cell histological subtype (ccRCC) represents the majority of RCC cases (~80%), followed by papillary and chromophobe, among other rarer subtypes [3]. The standard procedure for treatment of localized stages is surgery (partial or radical nephrectomy) [4]. However, approximately 30% of all RCC diagnoses occur at metastatic stages, requiring the introduction of systemic therapies due to the high risk of recurrence [1,3]. Targeting agents such as tyrosine kinase inhibitors (TKIs) have been the standard treatment for the management of metastatic disease with a reported benefit in the overall survival of patients [3]. The mechanism of action of these agents is based on the inhibition of the receptors of key regulatory signaling proteins that are crucial for cancer growth and invasion [5]. Sunitinib is the most used TKI for the management of metastatic RCC and acts by inhibiting vascular endothelial growth factor receptors (VEGFRs), platelet-derived growth factor receptors (PDGFR-α and β), and c-Kit kinase [6]. Pazopanib, more recently approved, has gained interest among the medical community due to the lower side effects observed in patients along with the reduced costs [7,8]. Its mechanism of action is based on blocking VEGFRs, PDGFRs, and c-Kit, as sunitinib, and also fibroblast growth factor receptors (FGFRs), IL-2-inducible T-cell kinase, and lymphocyte-specific protein tyrosine kinase [6,7,9]. These targeted cancer drugs have similar clinical efficacy, but some studies have reported differences in terms of tolerability [10,11] due to their activities on other kinases (commonly termed off-target effects) [12]. In fact, the two TKIs are associated with different patterns of toxicities as fatigue and hematologic toxicity after sunitinib treatment, while the liver function is more affected by pazopanib [7]. However, no recommendation has been made regarding the use of sunitinib *versus* pazopanib in first-line settings. Furthermore, the treatment paradigm for metastatic RCC has recently changed with the introduction of immune checkpoint inhibitors (ICI) in combination with VEGFR-TKIs [4,13,14], but the percentage of patients responding to this novel therapeutic strategy remains unsatisfactory (~10%) [15,16,17]. There are still no optimized guidelines for TKIs selection in these combination regimens to achieve the optimal response with minimum toxicity [15]. Thus, the identification of markers able to predict the efficacy and toxicity of these targeted drugs may overcome these issues, allowing the selection of the best therapeutic option for each patient.

In this way, pharmacometabolomics, a branch of the metabolomics field, has been used as a tool to inform about drug pharmacokinetics and pharmacodynamics which are extremely valuable for predicting the outcome (e.g., efficacy or toxicity) in an individual [18,19]. Hence, the analysis of the metabolome prior to and after a drug intervention represents an attractive approach to identify altered circulating metabolites that can elucidate about deviations on therapeutic responses [19]. Several in vitro pharmacometabolomic studies have been performed to understand the impact of TKIs on the metabolism of cancer cells, namely prostate cancer [20], breast cancer [20,21], colorectal cancer [21], hepatocellular carcinoma [22], and leukemia [23]. As concerns to RCC cells, only two studies have described the metabolic changes caused by sunitinib [24,25], and none for pazopanib. According to those studies, the main mechanisms of action of TKIs are related to dysregulations on important pathways involved in cell proliferation and growth, specifically lipid and amino acid metabolisms, but also in TCA cycle and glycolysis, which are the main sources of energy for cells and essential for tumor progression [26]. However, no studies have investigated the impact of TKIs on the metabolome of non-tumoral cell lines, which may be useful to evaluate the safety profile of those drugs. Moreover, few metabolomic studies have entailed the profiling of both intracellular (endometabolome) and extracellular (exometabolome) metabolites. Understanding changes in the extracellular compartment, in particular, may be a suitable strategy for identifying biomarkers for predicting and monitoring therapeutic responses in vivo since it may mimic more closely the metabolic changes in biofluids (e.g., blood, serum, urine) [27].

This study reports, for the first time to our knowledge, the impact of clinically relevant concentrations of sunitinib and pazopanib in the metabolic profiles of a metastatic RCC cell line (Caki-1) and a non-tumorigenic renal cell line (HK-2). This pharmacometabolomics approach, combined with the study of established traditional toxicological parameters, is herein used to provide novel insights into in vitro efficacy and safety of these drugs.

## 2. Results

### 2.1. Effects of Sunitinib and Pazopanib on Cell Viability, Cell Proliferation, Nuclear Morphological Characteristics, and Redox Status of Caki-1 and HK-2 Cells

**Figure 1:**
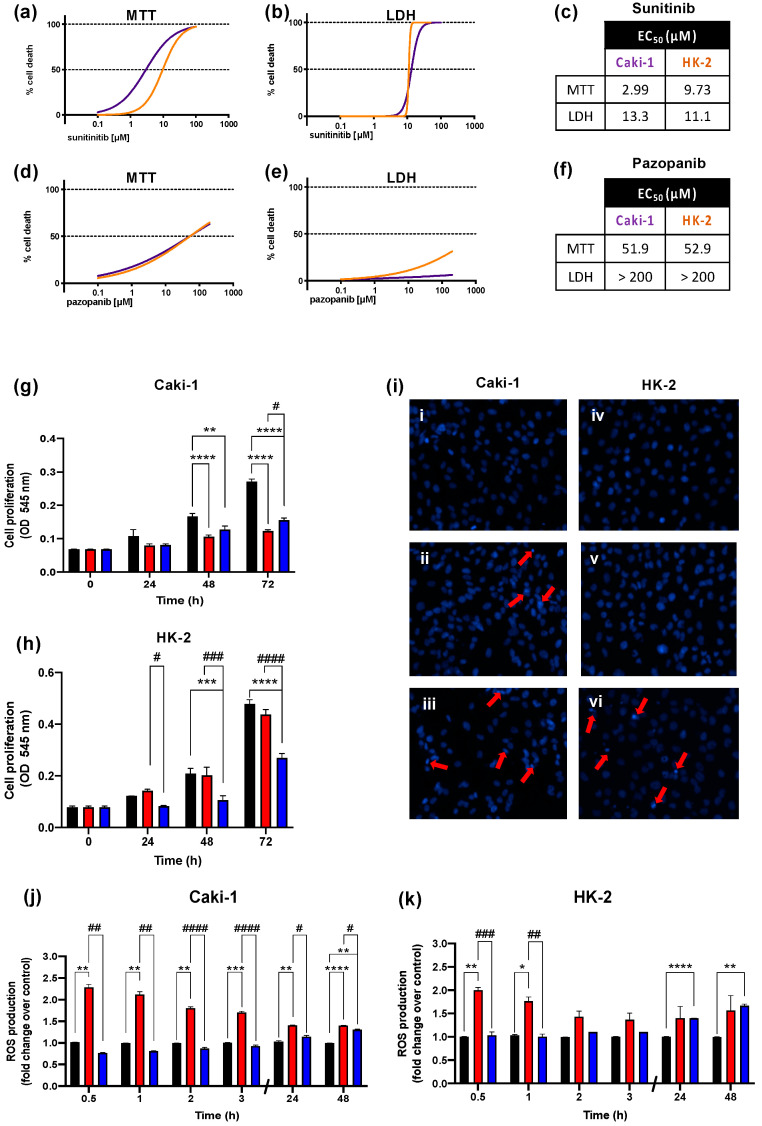
(**a**–**f**) Nonlinear regression models obtained for the cell death induced by sunitinib and pazopanib in Caki-1 (purple line) and HK-2 (orange line) cells, as evaluated by the MTT and LDH assays after 48 h exposure. (**g**,**h**) Inhibitory effect on proliferation of Caki-1 and HK-2 cells (black bars are non-exposed cells) exposed to 2 µM sunitinib (red bars) or 50 µM pazopanib (blue bars) after 24, 48, and 72 h, respectively. (**i**) Representative fluorescence microscopy images of Hoechst 33342/PI double staining of control (**i/i**) non-exposed Caki-1 cells, (**i/ii**) Caki-1 cells exposed to 2 µM sunitinib, (**i/iii**) Caki-1 cells exposed to 50 µM pazopanib, (**i/iv**) non-exposed HK-2 cells, (**i/v**) HK-2 cells exposed to 2 µM sunitinib, and (**i/vi**) HK-2 cells exposed to 50 µM pazopanib, for 48 h. Red arrows indicate early apoptotic cells (blue condensed nuclei). Original magnification ×200. (**j**,**k**) ROS production in Caki-1 and HK-2 cells (black bars are non-exposed cells) exposed to 2 µM sunitinib (red) or 50 µM pazopanib (blue) for 48 h, respectively. Results were obtained from three independent experiments, performed in triplicate, and are presented as mean ± standard error of the mean. * *p* < 0.05, ** *p* < 0.01, *** *p* < 0.001, and **** *p* < 0.0001 for sunitinib- or pazopanib-exposed cells vs. non-exposed cells. ^#^ *p* < 0.05; ^##^ *p* < 0.01; ^###^ *p* < 0.001; and ^####^ *p* < 0.0001 for pazopanib- vs. sunitinib- exposed cells.

In order to select the half maximal effective concentrations (EC_50_) for cytotoxic and metabolic experiments, a large range of concentrations of sunitinib (0.1, 0.2, 1, 2, 5, 10, 25, 50, and 100 µM) and pazopanib (0.1, 1, 5, 10, 25, 50, 100, and 200 µM) were considered to assess their effects on the viability of Caki-1 and HK-2 cell cultures through the MTT reduction and LDH leakage assays. Cells were also exposed to 0.25% DMSO (the maximum concentration used to prepare TKIs solutions) but no significant changes were observed (data not shown). Figure 1 shows the nonlinear regression models for the cell death obtained for both cell lines where it is possible to observe that the two TKIs caused a concentration-dependent loss of cell viability. The EC_50_ obtained for sunitinib was 2.99 µM (based on MTT assay, Figure 1a,c) and 13.3 µM (based on LDH assay, Figure 1b,c) for Caki-1 cells, and 9.73 µM (MTT, Figure 1a,c) and 11.06 µM (LDH, Figure 1b,c) for HK-2 cells. For pazopanib, the EC_50_ obtained in the MTT assay was 51.9 and 52.9 µM for Caki-1 and HK-2 cells, respectively (Figure 1d,f), but the EC_50_ based on the LDH assay (Figure 1e,f) could not be estimated from the concentration range tested. Of note, we were unable to test pazopanib concentrations above 200 μM due to solubility issues. Based on these results, a single concentration of sunitinib (2 µM) and pazopanib (50 µM) was chosen for the following experiments, corresponding to approximately 40–50% loss of cell viability, as measured by the MTT assay, but with no significant loss of membrane integrity, as indicated by the LDH assay. We then used the MTT assay to check how these concentrations affected the proliferation of Caki-1 and HK-2 cells. As shown in Figure 1g,h, both drugs had a significant inhibitory effect on the proliferation of Caki-1 cells (*p* ≤ 0.0001 and *p* ≤ 0.01 for sunitinib and pazopanib, respectively), reaching 55 and 43% inhibition for sunitinib and pazopanib after 72 h of incubation, respectively. On the other hand, only pazopanib significantly decreased cell growth of HK-2 cells (*p* ≤ 0.01 and *p* ≤ 0.001 for 48 and 72 h of exposure, respectively).

To investigate the cell death processes the Hoechst 33342/PI double staining was used to examine the nuclear morphological changes after 48 h of TKIs exposure. Representative fluorescence microscopy images are depicted in Figure 1i. Sunitinib-exposed Caki-1 cells exhibited chromatin condensation and brightly fluorescent pyknotic nuclei, with no red nuclear staining by PI, indicating early apoptotic events (Figure 1(i/ii)), whereas HK-2 cells exposed to sunitinib did not differ from control cells (Figure 1(i/v) and 1(i/iv), respectively). In contrast, pazopanib induced nuclei fragmentation and condensation in both Caki-1 and HK-2 cells (Figure 1(i/iii) and 1(i/vi), respectively). There was no PI staining, thus indicating that sunitinib and pazopanib did not cause necrosis in either cell line at the tested concentrations.

To determine the role of oxidative stress in the toxicity elicited by these drugs, ROS formation was measured in Caki-1 and HK-2 cells exposed to 2 μM sunitinib or 50 μM pazopanib at several timepoints up to 48 h. Figure 1j,k show that both drugs were capable to stimulate the production of ROS in both cell lines, albeit in different ways. Sunitinib elicited a significant increase in ROS production in Caki-1 cells at all tested timepoints, whereas HK-2 cells only showed a significant increase in ROS levels at early time points (0.5 and 1 h of exposure). On the other hand, pazopanib caused a weaker increase in ROS than sunitinib in both cell lines, which was statistically significant only at later timepoints (48 h in Caki-1 cells, 24 and 48 h in HK-2 cells).

### 2.2. Impact of Sunitinib and Pazopanib on the Endometabolome of Caki-1 and HK-2 Cells 

**Figure 2:**
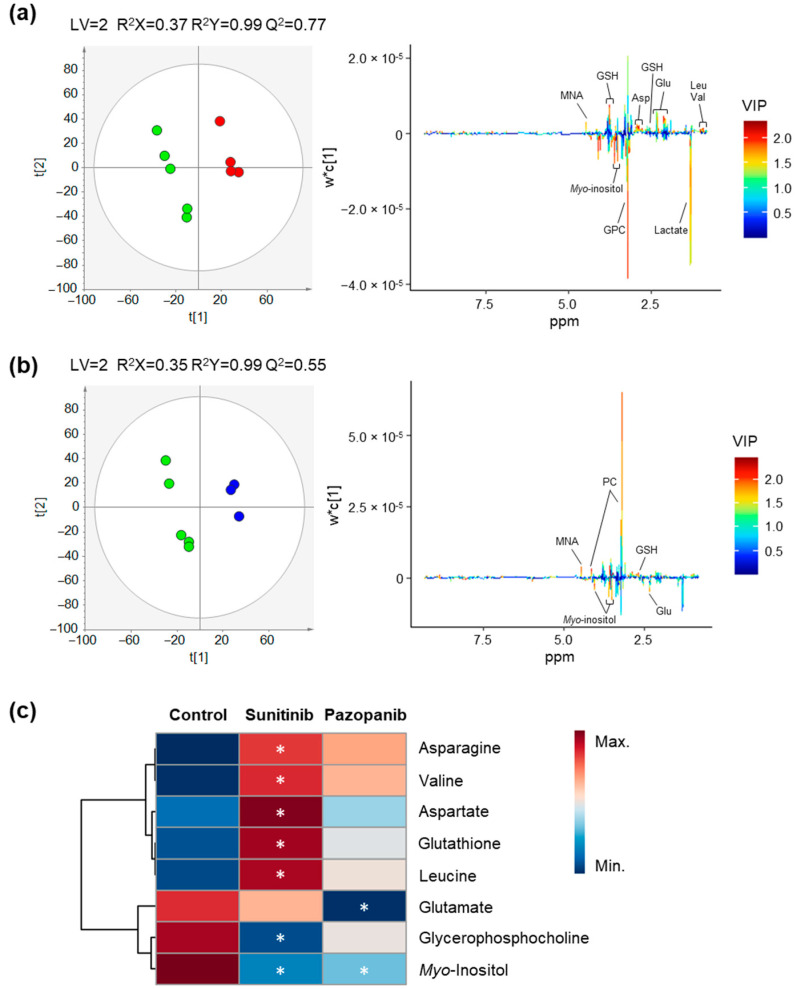
Impact of TKIs on the endometabolome (intracellular metabolites) profile of Caki-1 cells. (**a**,**b**) PLS-DA scores scatter plots (left) and loading plots (right) obtained for (**a**) non-exposed cells (green circles, *n* = 5) vs. cells exposed to 2 µM sunitinib (red circles, *n* = 4) and (**b**) non-exposed cells (green circles, *n* = 5) vs. cells exposed to 50 µM pazopanib (blue circles, *n* = 3). (**c**) Heatmap illustrating the mean levels of intracellular metabolites changing in Caki-1 cells after exposure with 2 µM sunitinib and 50 µM pazopanib. Rows correspond to the mean normalized peak area of each metabolite colored from minimum value (dark blue) to maximum value (dark red), while the columns represent each sample group. The control group represented in the first column corresponds to non-exposed cells. The statistical significance was assessed by comparison with the control cells (* *p* < 0.05). Abbreviations: Asp—aspartate, Glu—glutamate, GPC—glycerophosphocholine, GSH—glutathione, Leu—leucine, MNA–1—methylnicotinamide, PC—phosphocholine, Val—valine.

**Figure 3:**
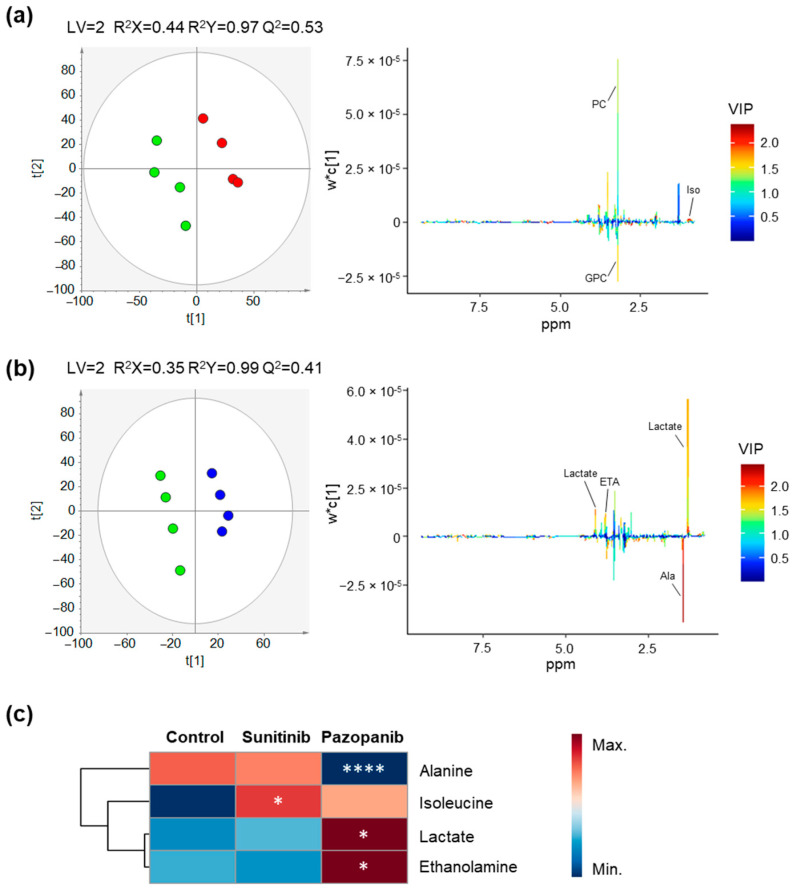
Impact of TKIs on the endometabolome (intracellular metabolites) profile of HK-2 cells. (**a**,**b**) PLS-DA scores scatter plots (left) and loading plots (right) obtained for (**a**) non-exposed cells (green circles, *n* = 4) vs. cells exposed to 2 µM sunitinib (red circles, *n* = 4) and (**b**) non-exposed cells (green circles, *n* = 4) vs. cells exposed to 50 µM pazopanib (blue circles, *n* = 4). (**c**) Heatmap illustrating the mean levels of intracellular metabolites changing in HK-2 cells after exposure with 2 µM sunitinib and 50 µM pazopanib. Rows correspond to the mean normalized peak area of each metabolite colored from minimum value (dark blue) to maximum value (dark red), while the columns represent each sample group. The control group represented in the first column corresponds to non-exposed cells. The statistical significance was assessed by comparison with the control cells (* *p* < 0.05, **** *p* < 0.0001). Abbreviations: Ala—alanine, ETA—ethanolamine, GPC—glycerophosphocholine, Iso—isoleucine, PC—phosphocholine.

A representative ^1^H NMR spectrum of the endometabolome of non-exposed (control) Caki-1 cells is shown in Figure S1. Overall, 37 metabolites were assigned including several amino acids, organic acids, nucleotides, sugars, among others. Complete metabolite annotations are provided in Table S1. Some alterations in metabolites levels were observed between the non-exposed Caki-1 and HK-2 cells indicating differences in amino acid (alanine, glutamate), arginine and proline (creatine and/or phosphocreatine), and glycerophospholipid (o-phosphocholine) metabolisms (Table S2). In addition, alterations on the levels of myo-inositol were detected, suggesting dysregulations on galactose metabolism, phosphatidylinositol signaling system or inositol phosphate metabolism between the two cell lines (Table S2). To investigate the effect of sunitinib and pazopanib on the endometabolome of Caki-1 and HK-2 cells, unsupervised and supervised pair-wise analyses were performed between exposed and non-exposed cells for each cell line. No apparent separation was observed in PCA models (data not shown), while PLS-DA models were able to discriminate exposed from non-exposed cells with good predictive power (Q2 > 0.4), as shown in Figure 2 and Figure 3 for Caki-1 and HK-2 cells, respectively. Based on the inspection of the corresponding loading plots, the magnitude and statistical significance of the variations was assessed as shown in Table 1 and in the heatmaps in Figure 2c and Figure 3c. In addition, the effect of DMSO (used as vehicle) on the endometabolome of both cells lines was investigated, but, as expected, no separation was obtained in either PCA or PLS-DA models when compared with non-exposed cells.

Overall, the sunitinib exposure induced more metabolic changes (seven altered metabolites) in Caki-1 cells than pazopanib (two altered metabolites) (Table 1). Sunitinib exposure caused a statistically significant decrease in the levels of glycerophosphocholine and myo-inositol, as well as a statistically significant increase in the levels of several amino acids and derivatives (asparagine, aspartate, leucine, valine, and glutathione). After pazopanib exposure, only a significant decrease in myo-inositol and glutamate levels was observed in the Caki-1 cells. Compared to tumoral cells, the endometabolome of HK-2 was less affected by both TKIs. Indeed, only a significant increase in the levels of isoleucine was observed after sunitinib exposure, whereas pazopanib exposure resulted in a significant decrease in alanine levels and a significant increase in ethanolamine and lactate levels.

### 2.3. Impact of Sunitinib and Pazopanib on the Exometabolome of Caki-1 and HK-2 Cells

**Figure 4:**
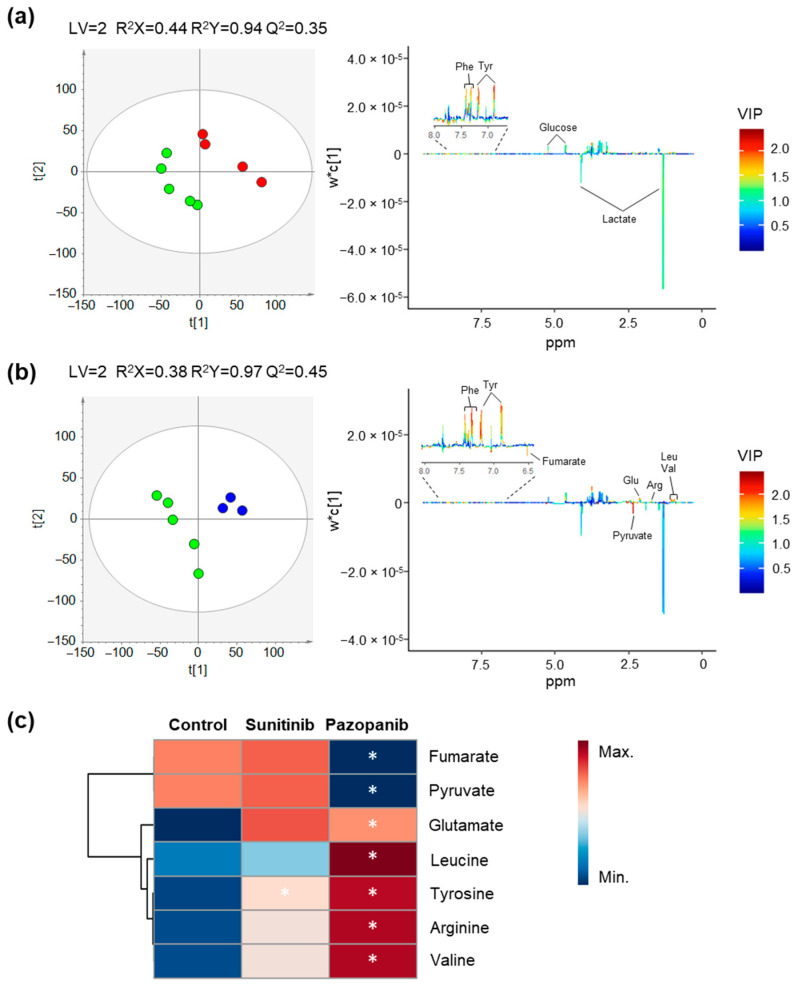
Impact of TKIs on the exometabolome (extracellular metabolites present in culture medium) profile of Caki-1 cells. (**a**,**b**) PLS-DA scores scatter plots (left) and loading plots (right) obtained for (**a**) non-exposed cells (green circles, *n* = 5) vs. cells exposed to 2 µM sunitinib (red circles, *n* = 4) and (**b**) non-exposed cells (green circles, *n* = 5) vs. cells exposed to 50 µM pazopanib (blue circles, *n* = 3). (**c**) Heatmap illustrating the mean levels of metabolites changing in culture media of Caki-1 cells after exposure with 2 µM sunitinib and 50 µM pazopanib. Rows correspond to the mean normalized peak area of each metabolite colored from minimum value (dark blue) to maximum value (dark red), while the columns represent each sample group. The control group represented in the first column corresponds to non-exposed cells. The statistical significance was assessed by comparison with the control cells (* *p* < 0.05). Abbreviations: Arg—arginine, Glu—glutamate, Leu—leucine, Phe—phenylalanine, Tyr—tyrosine, Val—valine.

**Figure 5:**
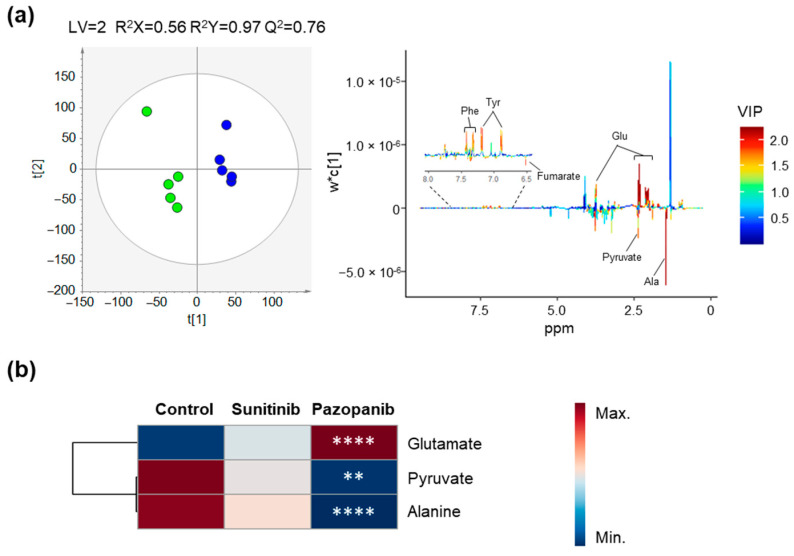
Impact of pazopanib on the exometabolome (extracellular metabolites present in culture medium) profile of HK-2 cells. (**a**) PLS-DA scores scatter plot (left) and loading plot (right) obtained for non-exposed cells (green circles, *n* = 5) vs. cells exposed to 50 µM pazopanib (blue circles *n* = 5). (**b**) Heatmap illustrating the mean levels of metabolites changing in culture media of HK-2 cells after exposure with 50 µM pazopanib. Rows correspond to the mean normalized peak area of each metabolite colored from minimum value (dark blue) to maximum value (dark red), while the columns represent each sample group. The control group represented in the first column corresponds to non-exposed cells. The statistical significance was assessed by comparison with the control cells (** *p* < 0.01, **** *p* < 0.0001). Abbreviations: Ala—alanine, Glu—glutamate, Phe—phenylalanine, Tyr—tyrosine.

The ^1^H NMR metabolic profiling of culture media of Caki-1 and HK-2 cells enabled the assignment of 33 metabolites from different chemical classes (e.g., amino acids, organic acids, and sugars). A representative ^1^H NMR spectrum of the culture medium of non-exposed (control) Caki-1 cells is shown in Figure S2 and the metabolites annotated are listed in Table S3. However, outside the scope of this study, we noticed statistically significant differences in the levels of metabolites participating in the tricarboxylic acid (TCA) cycle (lactate, succinate, formate, and glucose), glycerophospholipid (*o*-phosphocholine, *myo*-inositol), and amino acid (arginine, glutamine, glutamate) metabolisms between the extracellular culture media of non-exposed Caki-1 and HK-2 cells (Table S4). To investigate the metabolic alterations occurring in the culture media of Caki-1 and HK-2 cells after exposure with sunitinib and pazopanib, the same strategy described for endometabolome was applied for exometabolome data analysis. The results obtained are shown in Figure 4 and Figure 5, and Table 2.

The PLS-DA scores scatter plots obtained for Caki-1 exometabolome (Figure 4) showed a reasonable separation between the extracellular culture media of exposed and non-exposed cells (Q^2^ ≥ 0.35). After inspection of the corresponding loading plots, the magnitude of variation and statistical significance of discriminant metabolites was assessed and is presented in Table 2 and the heatmap of Figure 4c. No separation was observed in the PCA and PLS-DA models of Caki-1 and HK-2 cells exposed to DMSO (vehicle) compared with controls (non-exposed cells). The exposure of Caki-1 cells to sunitinib unveiled a significant alteration of only one extracellular metabolite, whereas seven compounds were found significantly altered after pazopanib exposure. The relative comparison with blanks (culture medium without cells) allowed the interpretation of metabolite alterations in terms of excretion (E) or consumption (C) as indicated in Table 2 and shown in the boxplots of Figure S3. Thus, sunitinib exposure was only responsible for a significant lower consumption of tyrosine in Caki-1 cells. Contrastingly, the significant alterations found on the exometabolome of Caki-1 cells after exposure with pazopanib comprised a significant lower consumption of arginine, leucine, tyrosine, and valine, as well as a significantly higher excretion of glutamate, and a lower excretion of fumarate and pyruvate in Caki-1 cells exposed with pazopanib. 

Regarding the impact of both TKIs on the exometabolome profile of the non-tumoral renal (HK-2) cells, no discrimination was observed for sunitinib exposure (data not shown), whereas a clear separation with high predictive power (Q^2^ = 0.76) was observed for pazopanib (Figure 5). The alterations in the exometabolome of HK-2 cells exposed to pazopanib included a significant lower excretion of pyruvate and alanine, and a significant higher excretion of glutamate, as listed in Table 2 and shown in the boxplots of Figure S3.

## 3. Discussion

The effects of two TKIs commonly used in the treatment of metastatic RCC, sunitinib and pazopanib, were investigated on the cellular and molecular responses of both tumoral and non-tumoral human renal cells with the goal of providing new insights into their efficacy and safety. Importantly, the cytotoxicity and metabolic effects were investigated by exposing cells to near clinically relevant concentrations of the two drugs (2 µM sunitinib and 50 µM pazopanib, corresponding to EC_40_–EC_50_ obtained in the MTT assay). Indeed, plasma concentrations of 0.3–0.4 and 40 μM were found in patients receiving sunitinib and pazopanib treatment, respectively [28,29]. Despite lower plasmatic concentrations have been detected after sunitinib treatment, several authors have reported that this drug is sequestered into the lysosomal compartments of tumour cells, with intratumoral concentrations reaching up to 10 μM [25,28,30].

**Figure 6:**
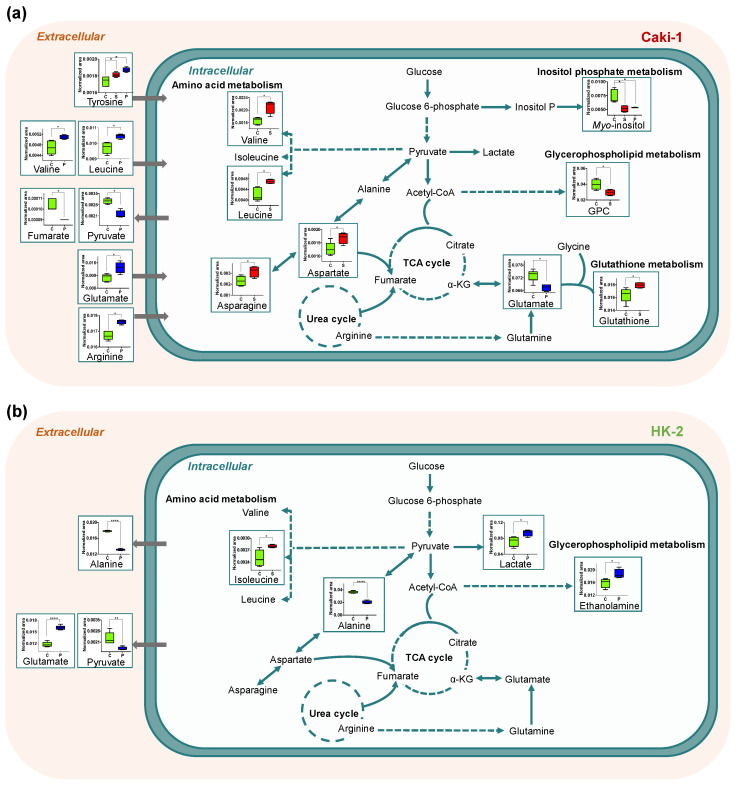
Overview of the intracellular and extracellular metabolic alterations occurring in (**a**) metastatic RCC cells (Caki-1) and (**b**) non-tumoral renal cells (HK-2) after exposure to 2 µM sunitinib (S, red) or 50 µM pazopanib (P, blue) compared with the corresponding controls (C, green) (* *p* < 0.05, ** *p* < 0.01, **** *p* < 0.0001). The arrows indicate metabolites consumed (→) and excreted (←) considering the non-exposed cells (control) compared with blanks. GPC: glycerophosphocholine.

Our findings unveiled important differences in the toxicity and metabolic responses induced by these TKIs. Sunitinib showed selective toxicity against target cancer cells, inhibiting cell growth and prompting apoptotic cell death in Caki-1 cells, while having no effect on non-tumoral renal cells. In line with this, the metabolic dysregulations caused by sunitinib exposure were more pronounced in tumoral cells, with only a minor significant change (increase intracellular levels of isoleucine) in non-tumoral renal cells. On the other hand, pazopanib inhibited proliferation and induced early apoptosis in a similar way in both cell lines, along with several metabolic dysregulations. An overview of the intracellular and extracellular metabolic alterations observed in Caki-1 and HK-2 cells after exposure with the two TKIs under study, as well as their possible association with biochemical pathways, is presented in Figure 6 and is discussed in this section.

Overall, the estimated EC_50_ values for the LDH leakage assay were higher than those observed in the MTT reduction for both TKIs in either cell line. As marker of cell membrane integrity, these data are in agreement with the predominance of apoptotic (and mitochondrion-dependent) cell death over necrosis, which was in fact absent in the H33342/PI double staining at clinically relevant concentrations of the TKIs. The potential of sunitinib to induce apoptosis in RCC cells, in addition to its antiangiogenic effects, has previously been demonstrated in vitro [31,32]. One of these studies [31] compared the cellular effects of sunitinib and pazopanib revealing that sunitinib was able to induce a direct apoptotic effect (cytotoxic effect), whereas pazopanib only inhibited cellular proliferation (cytostatic effect) in different RCC cell lines. In agreement with this study, we showed that sunitinib has a higher cytotoxic potential. However, at the relevant and equipotent concentrations selected for this study, pazopanib triggered apoptotic cell death in both tumoral and non-tumoral cells, which is consistent with the similar EC_50_ values found for this TKI in the MTT reduction assay. On the other hand, apoptotic events resulting from exposure to sunitinib were only noticeable in Caki-1 cells. Our data further demonstrate that both TKIs detain cytostatic potential in tumoral cells at levels at which there is no significant membrane rupture (i.e., no substantial increase in LDH leakage), but only pazopanib was capable of significantly inhibiting proliferation of HK-2 cells. Together, these results support a more selective and safer profile for sunitinib. Importantly, because there is no cell membrane rupture at the concentrations used in this study, we are investigating early mechanisms of cell response.

Regarding the impact of sunitinib on the cell metabolome, this TKI caused a significant increase in the intracellular levels of various amino acids (leucine, valine, aspartate, and asparagine) in metastatic RCC cells, indicating amino acid metabolism dysregulation and/or possible intracellular accumulation of these amino acids. Remarkably, a lower consumption of tyrosine was observed in the extracellular medium of Caki-1 cells exposed to sunitinib. This alteration may be explained by a slowdown in protein synthesis caused by sunitinib, resulting in an accumulation of this metabolite in the extracellular medium, which is expected due to the inhibition of cell proliferation caused by sunitinib. In agreement, several studies have reported an upregulation in the levels of amino acids in lung and triple-negative breast cancer cells after exposure with another TKI (erlotinib) [33,34]. Furthermore, a significant decrease in intracellular glycerophosphocholine and *myo*-inositol levels was observed in Caki-1 cells, which may also be due to the inhibition of cell proliferation induced by sunitinib. These metabolites participate in the glycerophospholipid and inositol phosphate metabolisms, which are crucial pathways for the biogenesis of cell membranes and signaling processes in tumor cell proliferation [35,36], respectively. Moreover, sunitinib had a much lower effect on the non-tumoral renal cell line HK-2, with only one intracellular metabolite (isoleucine) found dysregulated. Hence, with the lower effects observed in the inhibition of cell proliferation and absence of alterations on nuclear morphology of HK-2 cells, our results enhance the target selectivity for sunitinib. 

Concerning the impact of pazopanib on cell metabolome, our findings unveiled that the intracellular composition of Caki-1 was less affected by pazopanib than for sunitinib. Similarly to sunitinib, there was only a significant decrease in the levels of *myo*-inositol in Caki-1 cells after pazopanib exposure. Due to its involvement in several signaling processes important for cell proliferation and migration, *myo*-inositol has been established as an important growth-promoting factor of mammalian cells [36,37]. Therefore, the deprivation of inositol forms, referred also as nutrients for cells, can induce oxidative stress in the cellular environment leading to the activation of autophagy [38]. Indeed, previous research has linked sunitinib and pazopanib to autophagy via apoptosis [39,40], which was also observed in our study after 48 h of exposure. Taken together, our results suggest that the downregulation of this metabolite may be a marker of TKI efficacy. According to the number of altered metabolites, we observed a greater impact of pazopanib exposure on the exometabolome of Caki-1 cells. The alterations included a significantly lower consumption of amino acids, which is consistent with a slowdown in protein synthesis. It is worth noting that both sunitinib and pazopanib exposure led to a significantly lower consumption of tyrosine levels from extracellular medium of Caki-1 cells. Furthermore, unlike sunitinib, pazopanib caused significant changes in several intracellular and extracellular metabolites in HK-2 cells, but its impact on the exometabolome was more pronounced, as previously observed in Caki-1 cells, including alterations in metabolites participating in energy metabolism (pyruvate) and cellular antioxidant capacity (glutamate). Remarkably, non-tumoral HK-2 cells had dramatically lower intracellular and extracellular levels of alanine after exposure to pazopanib. Alanine aminotransferase (ALT) is an enzyme that catalyzes the reversible transfer of the amino group from alanine to 2-oxoglutarate to form glutamate and pyruvate, which is used for cellular energy production [41]. Apart from liver cells, this enzyme is also found in other main organs such as the kidney [42]. Thus, our results suggest that HK-2 cells use the intracellular and extracellular alanine to produce pyruvate while the excess of generated glutamate is excreted. Supporting this hypothesis, the expression of ALT was found increased in patients treated with pazopanib compared to those treated with sunitinib [7].

The oxidative stress mediated by both TKIs in Caki-1 and HK-2 cells was also distinct. ROS are reactive oxygen-containing molecules that are produced mainly in mitochondria, but also in peroxisomes and endoplasmic reticulum through β-oxidation of fatty acids and oxidation of proteins, respectively. Moderate levels of ROS are required for cellular homeostasis since they are involved as regulators in several cellular functions [43]. ROS production is typically elevated in cancer cells to support cell proliferation and tumor progression, but also to participate in xenobiotic defense [43,44,45]. Recent research indicates that some chemotherapeutics exert their therapeutic action by increasing intracellular ROS levels that exceed the antioxidant capacity of cancer cells, resulting in cell death via apoptosis and autophagy [6,44] with better clinical outcomes for cancer patients [46]. In line with this, our results demonstrated that sunitinib prompted a robust early increase in ROS formation in both Caki-1 and HK-2 cells (respectively, 2.3- and 2.0-fold increase over control at the first recorded timepoint, 30 min). This effect was more marked in the tumoral cell line, where ROS levels remained significantly elevated even after 48 h of exposure. In agreement, previous in vitro studies have found that sunitinib cause mitochondrial damage and intracellular ROS accumulation in cardiomyocytes and hepatocytes [47,48,49]. The metabolomic analysis revealed a significant increase in intracellular glutathione levels in Caki-1 cells exposed to sunitinib, implying activation of the glutathione antioxidant system, the primary cellular detoxification mechanism, to counteract the increased oxidative stress caused by this TKI [47,50]. Sunitinib, according to our findings, also decreases tumor cell viability and growth, and induces early apoptotic cell death in Caki-1 cells. Taken together, the efficacy of this TKI appears to be related to an early increase in oxidative stress in RCC cells, resulting in cell death via apoptosis and/or autophagy, as previously reported [6,50]. No statistically significant differences in ROS formation were observed with pazopanib at the initial timepoints, but a significant increase was observed after 48 h of exposure (1.4-fold increase over control). Nonetheless, pazopanib-exposed Caki-1 cells exhibited decreased intracellular levels of glutamate, which may be related with its use for glutathione synthesis [51] that is required to prevent ROS accumulation [52]. However, glutathione was not found statistically altered in Caki-1 as occurred with sunitinib, which is in line with the observed lower and delayed potential of pazopanib to trigger ROS production in Caki-1 cells. More recently, it was found that the hepatic metabolism of pazopanib leads to the formation of several reactive metabolites such as cysteine conjugates, aldehyde derivatives and *N*-oxide metabolites [53], which can induce oxidative stress. This may explain the toxic potential of this TKI in both tumoral and non-tumoral cells [53]. In fact, our results demonstrated a tendency for higher oxidative stress over time, measured by statistically significant elevated ROS formation at 24 and 48 h after pazopanib exposure in the non-tumoral cell line HK-2.

One limitation of our study is the different sample size (*n* = 3 to 5) considered in statistical analysis of the endometabolome and exometabolome of Caki-1 and HK-2 cells, since some samples had to be excluded due to poor signal-to-noise ratio (S/N) in the ^1^H NMR spectra. In addition, the study of other RCC cell lines as well as the combination of metabolomics with other -omics approaches (e.g., proteomics) would enrich the study. Despite these limitations, we believe that we have selected the most representative cell lines available, namely a metastatic RCC cell line (Caki-1) and a non-tumoral human renal cell line (HK-2). Moreover, this study combined traditional toxicity assays with metabolomics and we also extended for the first time, to our knowledge, the study of the impact of sunitinib and pazopanib on both intracellular and extracellular compartments. We believe that these findings can contribute to improve the treatment guidelines for metastatic RCC. A preference for the use of pazopanib has been referred [8] due to its reduced costs, but our study reveals that sunitinib exhibits higher selectivity for tumoral cells in its mechanism of action and a lower level of renal toxicity. This study emphasizes the importance of in vitro studies to address the alterations at the cellular level avoiding the confounding factors present in the in vivo matrices. 

## 4. Materials and Methods

### 4.1. Cell Culture Materials and Chemicals

RPMI-1640 medium was obtained from Sigma-Aldrich (Sigma-Aldrich, St. Louis, MO, USA). The antibiotic mixture penicillin/streptomycin (10.000 U/mL/10.000 mg/mL), heat-inactivated fetal bovine serum (FBS) and trypsin-EDTA (0.25%) were purchased from GIBCO Invitrogen (GIBCO, Barcelona, Spain). Hydrochloric acid (HCl), sodium hydrogen carbonate and Hank′s Balanced Salt Solution (HBSS) were obtained from Merck (Merck, Darmstadt, Germany). Deuterium oxide (D_2_O) was provided by Eurisotop (Eurisotop, Saint-Aubin, France) and D_2_O containing 0.05 wt% 3-(trimethylsilyl)propionic-2,2,3,3-d4 acid (TSP) sodium salt was purchased from Sigma-Aldrich (Sigma-Aldrich, St. Louis, MO, USA). Thiazolyl blue tetrazolium bromide (MTT), sodium pyruvate, β-nicotinamide adenine dinucleotide reduced disodium salt hydrate (β-NADH), β-nicotinamide adenine dinucleotide 2′-phosphate reduced tetrasodium salt hydrate (β-NADPH), and dichlorodihydrofluorescein diacetate (DCFH-DA) were purchased from Sigma-Aldrich (Sigma-Aldrich, St. Louis, MO, USA). Solvents such as dimethyl sulfoxide (DMSO), methanol and chloroform were acquired from Sigma-Aldrich (Sigma-Aldrich, St. Louis, MO, USA). DMSO cell culture grade was obtained from PanReac (PanReac, Barcelona, Spain). Sunitinib malate (≥98%) and pazopanib (≥98%) were purchased from Sigma-Aldrich (Sigma-Aldrich, St. Louis, MO, USA).

### 4.2. Cell Lines and Culture Conditions

The human metastatic ccRCC cell line (Caki-1) and the non-malignant renal epithelial cell line (HK-2) were obtained from the American Type Culture Collection (ATCC; Manassas, VA, USA). Both cell lines were grown in RPMI-1640 supplemented with 10% of FBS and 1% penicillin/streptomycin and incubated in a humidified incubator (37 °C with 5% CO_2_). After an adaptation stage of at least three passages, the experiments were carried out within the passages 11 to 16 for Caki-1 and 11 to 17 for HK-2. Additionally, they were routinely tested for *Mycoplasma* spp. contamination (TaKaRa PCR Mycoplasma Detection Set, Clontech Laboratories, Mountain View, CA, USA).

#### 4.2.1. Cell Viability and Proliferation Assays

To assess the effects of the two TKIs on cell viability, Caki-1 and HK-2 cells were seeded in 96-well plates at an initial density of 7.5 × 10^4^ and 5 × 10^4^ cells/mL, respectively, and incubated on the following day with sunitinib (concentration range of 0.1–100 µM) or pazopanib (concentration range of 0.1–200 µM) in a humidified incubator (37 °C with 5% CO_2_). The effects on cell viability were evaluated after 48 h of drug exposure through MTT reduction and LDH leakage assays according to previous protocols [54]. The experiments were repeated in triplicate in at least 7 independent experiments for MTT assays and 4 independent assays for LDH assays. 

The inhibitory effect of sunitinib and pazopanib on cell proliferation was determined by seeding Caki-1 and HK-2 cells in 96-well plates at an initial density of 4 × 10^4^ cells/mL and 3 × 10^4^ cells/mL, respectively. After growing overnight, cells were exposed to 2 µM sunitinib or 50 µM pazopanib (EC_40_–EC_50_ values obtained from MTT cytotoxicity assay) for 24, 48, and 72 h in a humidified incubator (37 °C with 5% CO_2_). The MTT reduction assay was performed at the end of each exposure time. All experiments were performed in triplicate in at least three independent assays for each time point of analysis.

#### 4.2.2. Measurement of Intracellular Reactive Oxygen Species

Intracellular production of reactive oxygen species (ROS) was monitored via the dichlorodihydrofluorescein diacetate (DCFH-DA) fluorescence assay as described elsewhere [54]. Caki-1 and HK-2 were seeded in 96-well plates at the initial density of 7.5 × 10^4^ cells/mL and 5 × 10^4^ cells/mL, respectively, and, after overnight cell adhesion, cells were incubated at 37 °C with DCFH-DA for 30 min protected from light. The supernatant was then rejected, and the cells were exposed to 2 µM sunitinib or 50 µM pazopanib for 48 h in a humidified incubator (37 °C with 5% CO_2_). ROS production was measured at the following timepoints: 0.5, 1, 2, 3, 24, and 48 h. A total of three independent assays were performed in triplicate and data were normalized to control (non-exposed) cells.

#### 4.2.3. Assessment of Nuclear Morphological Changes with Hoechst 33342/PI Fluorescent Staining

For characterization of cell death processes, Caki-1 and HK-2 cells were seeded in 24-well plates (initial density of 10 × 10^4^ cells/mL and 7.5 × 10^4^ cells/mL, respectively), let to attach overnight, and then exposed to 2 µM sunitinib or 50 µM pazopanib for 48 h, at 37 °C with 5% CO_2_. At the end of the incubation period, cells were washed with HBSS, incubated with 50 μM propidium iodide (PI) for 15 min, washed again with HBSS, and incubated with 5 μg/mL Hoechst 33342 solution for 5 min. The fluorescence was observed using a fluorescent microscope and the images were captured with the Lionheart FX Automated Microscope (Biotek Instruments Inc., Winooski, VT, USA).

### 4.3. Metabolite Extraction and Sample Preparation for ^1^H NMR-Based Metabolomics

For ^1^H NMR metabolic studies, cell lines were seeded in 60 mm cell culture dishes at an initial density of 22.5 × 10^4^ cells/mL. After 24 h, the medium was changed to fresh medium, and the following conditions were prepared: controls (non-exposed cells), vehicle (exposure with 0.25% DMSO, considering the maximum concentration of DMSO used to prepare the TKIs solutions), exposed cells (2 µM sunitinib or 50 µM pazopanib) and culture medium without cells (blanks), and incubated at 37 °C with 5% CO_2_ for 48 h. At the end of the incubation period, blank and exposure media were collected for exometabolome analysis, and adherent cells were collected for endometabolome analysis. The exposure media were centrifuged (1200× *g*, 5 min, 4 °C), and the supernatant stored at −80 °C until ^1^H NMR analysis. The cell monolayers were washed twice with PBS before intracellular metabolites extraction using a dual phase extraction method (methanol/chloroform/water 4:4:2.85) adapted from the literature [55]. Briefly, 800 µL of cold methanol was added to stop cellular metabolism and the cells were scrapped and transferred to a microcentrifuge tube containing 0.5 mm glass beads to facilitate cell breakage using the vortex for 2 min. Then, 320 µL of chloroform (added twice) and 320 µL of water were added (the samples were vortexed 2 min between each solvent addition). After 10 min of rest on ice, the samples were centrifuged at 2000× *g* for 15 min plus 10,000× *g* for 2 min at 4 °C. The upper phase containing the polar metabolites was collected, lyophilized, and stored at −80 °C until analysis. At least three independent experiments from different passages were performed considering one sample for each condition under study.

For NMR analysis, 585 µL of culture medium were mixed with 65 µL of deuterium oxide (D_2_O) containing 0.25% TSP, centrifuged at 10,410× *g* for 5 min at 4 °C, and 600 µL of the supernatant were transferred to a 5 mm NMR tube. The dried extracts were resuspended in 650 µL of D_2_O, centrifuged at 10,410× *g* for 5 min at 4 °C, and 600 µL of the supernatant were transferred to a 5 mm NMR tube.

### 4.4. ^1^H NMR Analysis and Metabolite Annotation

The ^1^H NMR experiments were performed on a Bruker Avance III HD 600 MHz spectrometer (Bruker BioSpin, Rheinstetten, Germany) equipped with a cryoprobe at 26.85 °C (300 K). ^1^H standard 1D spectra (*noesypr1d*) were recorded for dried intracellular extracts and culture medium supernatant with 4 s relaxation delay, 100 ms mixing time, 256 transients, 64k complex data points, 10,080.646 Hz spectral width and 3.25 s acquisition time. 1D spectra were processed with a 1.0 Hz exponential line-broadening function, manually phased and baseline corrected, and chemical shifts referenced to lactate at δ = 1.32 ppm for intracellular extracts and glucose at δ = 5.23 ppm for culture medium. 2D NMR experiments, namely total correlation spectroscopy (TOCSY) and heteronuclear single quantum coherence (HSQC) spectra were also recorded for selected control samples (intracellular extract and culture medium of non-exposed cells) to assist in metabolite annotation. In addition, the ^1^H resonances present in the samples were compared with the ^1^H NMR spectra of standard compounds existing in the Biological Magnetic Resonance Bank [56] and the Chenomx NMR suite 8.4 software (Chenomx Inc., Edmonton, AB, Canada). 

### 4.5. Data Pre-Processing

Prior to statistical analysis, the ^1^H NMR spectra were uploaded to the NMRProcFlow 1.4 online tool [57] for spectral data pre-processing. For intracellular extracts, the spectral regions of residual water (5.20–4.67 ppm), ethanol (1.28–1.13 ppm and 3.67–3.62 ppm), methanol (3.36–3.34), and DMSO (2.74–2.69) were excluded from the whole spectral region between 9.35–0.75 ppm. For culture medium, the spectral regions of residual ethanol (1.21–1.13 and 3.68–3.63 ppm), DMSO (2.86–2.56 ppm), and water (5.03–4.67 ppm) were excluded from the whole spectral region between 10.0–0.25 ppm. Then, the spectra of intracellular extracts and culture medium were aligned using the Parametric Time Warping method [58], followed by bucketing with uniform spectral width (0.001 ppm, signal to noise ratio = 3) and normalization by the total area (TA). 

### 4.6. Statistical Analyses

Nonlinear regression curves obtained through MTT and LDH assays were constructed and analyzed using the GraphPad Prism (Version 8.2.1, San Diego, CA, USA). For analysis of antiproliferative effects (obtained using the MTT assay) and oxidative stress (obtained by the measurement of ROS levels), results were expressed as mean ± standard error of the mean and the statistical analysis was performed using the GraphPad Prism (Version 8.2.1, San Diego, CA, USA). Multiple comparisons were performed through two-way ANOVA test, followed by Tukey′s post hoc test. The results were considered statistically significant when *p* < 0.05 (confidence level 95%).

For interpretation of ^1^H NMR data, the final matrices were scaled to unit variance (UV) and analyzed through multivariate analysis methods such as principal component analysis (PCA) and partial least squares discriminant analysis (PLS-DA) in SIMCA 13 (Umetrics, Umea, Sweden). A default sevenfold internal cross-validation was used to confirm the performance of the PLS-DA models, retrieving the number of latent variables (LV), the fraction of X explained variance (R^2^X), the fraction of Y explained variance (R^2^Y), and the fraction of Y variation predicted by the X model or predictive ability (Q^2^). The PLS-DA loadings plots were created in R 4.0.3 software [59] using the ggplot2 package [60]. Relevant resonances identified in PLS-DA loadings plots with variable importance for the projection (VIP) higher than 1.0 were considered for *t*-test and Mann–Whitney test (GraphPad Prism 8.2.1, San Diego, CA, USA). *p* Values below 0.05 were considered statistically significant. Effect size (ES) and the corresponding standard error (SE) [61] were also computed for statistically significantly different metabolites.

## 5. Conclusions

Overall, our combined approach of traditional toxicity endpoints and metabolomics evidenced distinct patterns of in vitro safety and selectivity of sunitinib and pazopanib, which are two TKIs widely used in the treatment of metastatic RCC. Sunitinib showed a greater impact on metastatic renal tumoral cells, both in terms of cytotoxicity (reduction on cell proliferation, induction of oxidative stress and cell death) and metabolic dysregulations (mainly in the intracellular compartment), with no substantial effects on non-tumoral renal cells. Contrastingly, pazopanib triggered similar cytotoxic and metabolic effects on both cell types. These findings suggest that sunitinib may detain better tumor selectivity and lesser nephrotoxic potential when compared to pazopanib. Further studies should be performed to investigate if the fluctuations in metabolite levels induced by sunitinib and pazopanib can be candidate biomarkers to monitor in vivo therapeutic efficacy and safety.

## Figures and Tables

**Table 1:** List of metabolites significantly altered in the endometabolome of Caki-1 and HK-2 cells after exposure with 2 µM sunitinib or 50 µM pazopanib in comparison with the corresponding non-exposed cells (control).

Class/Metabolite	Caki-1 Cells				HK-2 Cells				Dysregulated Metabolic Pathways
	2 µM Sunitinib		50 µM Pazopanib		2 µM Sunitinib		50 µM Pazopanib		
	ES ± SE	*p* Value	ES ± SE	*p* Value	ES ± SE	*p* Value	ES ± SE	*p* Value	
*Amino acids and derivatives*									
Alanine	-	-	-	-	-	-	−6.80 ± 3.55	<0.0001	Aminoacyl-tRNA biosynthesis
Asparagine	1.85 ± 1.45	0.0292	-	-	-	-	-	-	Aminoacyl-tRNA biosynthesis
Aspartate	1.77 ± 1.43	0.0340	-	-	-	-	-	-	Aminoacyl-tRNA biosynthesis
Glutamate	-	-	−2.44 ± 1.73	0.0145	-	-	-	-	Aminoacyl-tRNA biosynthesis, Glutathione metabolism
Glutathione	1.71 ± 1.41	0.0317	-	-	-	-	-	-	Glutathione metabolism
Leucine	2.49 ± 1.64	0.0159	-	-	-	-	-	-	Aminoacyl-tRNA biosynthesis
Valine	2.78 ± 1.74	0.0159	-	-	-	-	-	-	Aminoacyl-tRNA biosynthesis
Isoleucine	-	-	-	-	2.03 ± 1.56	0.0289	-	-	Aminoacyl-tRNA biosynthesis
*Amino alcohols*									
Ethanolamine	-	-	-	-	-	-	1.93 ± 1.53	0.0348	Glycerophospholipid metabolism
*Glycerophospholipids*									
Glycerophosphocholine	−1.87 ± 1.45	0.0317	-	-	-	-	-	-	Glycerophospholipid metabolism
*Organic acids*									
Lactate	-	-	-	-	-	-	1.97 ± 1.54	0.0324	Energy metabolism
*Sugars*									
*Myo*-inositol	−2.19 ± 1.55	0.0159	−1.96 ± 1.57	0.0357	-	-	-	-	Galactose metabolism, inositol phosphate metabolism, phosphatidylinositol signaling system

ES: effect size, SE: standard error. Statistically significance assessed using *t*-test and Mann–Whitney test.

**Table 2:** List of metabolites significantly altered in the exometabolome (culture medium) of Caki-1 and HK-2 cells after exposure with 2 µM sunitinib or 50 µM pazopanib in comparison with the corresponding non-exposed cells (control).

Class/Metabolite	Caki-1 Cells				HK-2 Cells		Dysregulated Metabolic Pathways
	2 µM Sunitinib		50 µM Pazopanib		50 µM Pazopanib		
	ES ± SE	*p* Value	ES ± SE	*p* Value	ES ± SE	*p* Value	
*Amino acids and derivatives*							
Alanine	-	-	-	-	−22.7 ± 10.0 ^E^	<0.0001	Aminoacyl-tRNA biosynthesis
Arginine	-	-	3.42 ± 2.09 ^C^	0.0357	-	-	Aminoacyl-tRNA biosynthesis
Glutamate	-	-	2.03 ± 1.59 ^E^	0.0353	7.16 ± 3.33 ^E^	<0.0001	Aminoacyl-tRNA biosynthesis, glutathione metabolism
Leucine	-	-	2.31 ± 1.68 ^C^	0.0357	-	-	Aminoacyl-tRNA biosynthesis
Tyrosine	1.58 ± 1.38 ^C^	0.0476	2.56 ± 1.77 ^C^	0.0357	-	-	Aminoacyl-tRNA biosynthesis
Valine			1.78 ± 1.52 ^C^	0.0357	-	-	Aminoacyl-tRNA biosynthesis
*Organic acids*							
Fumarate	-	-	−3.04 ± 1.94 ^E^	0.0179	-	-	Energy metabolism
Pyruvate	-	-	−3.88 ± 2.27 ^E^	0.0357	−2.27 ± 1.50 ^E^	0.0075	Energy metabolism

ES: effect size, SE: standard error. Statistically significance assessed using *t*-test or Mann–Whitney test. ^E, C^ Metabolites excreted and consumed when compared with blanks (cell culture medium without cells).

## Data Availability

Data available upon request.

## Supplementary Materials

The following supporting information can be downloaded at: https://www.mdpi.com/article/10.3390/ijms23179898/s1.
