# Supplementary material for: Sunitinib versus Pazopanib Dilemma in Renal Cell Carcinoma: New Insights into the In Vitro Metabolic Impact, Efficacy, and Safety
Source: Int J Mol Sci. 2022 Aug 31;23(17):9898. doi: 10.3390/ijms23179898 (PMC9456255; doi:10.3390/ijms23179898)
Supplement: Supplementary file 1 [file ijms-23-09898-s001.zip › ijms-1884123-supplementary.pdf]

International Journal of
Molecular Sciences

MDPI

Article

Sunitinib versus Pazopanib Dilemma in Renal Cell Carcinoma: New Insights into the In Vitro Metabolic Impact, Efficacy, and Safety

Filipa Amaro [1,2,*], Carolina Pisoeiro [1,2], Maria João Valente [3], Maria de Lourdes Bastos [1,2], Paula Guedes de Pinho [1,2], Márcia Carvalho [1,2,4,5] and Joana Pinto [1,2,*]

1 Associate Laboratory i4HB, Department of Biological Sciences, Laboratory of Toxicology, Faculty of Pharmacy, University of Porto, 4050-313 Porto, Portugal
2 UCIBIO-REQUIMTE, Department of Biological Sciences, Laboratory of Toxicology, Faculty of Pharmacy, University of Porto, 4050-313 Porto, Portugal
3 National Food Institute, Technical University of Denmark, Kongens Lyngby, 2800 Copenhagen, Denmark
4 FP-I3ID, FP-BHS, University Fernando Pessoa, 4200-150 Porto, Portugal
5 Faculty of Health Sciences, University Fernando Pessoa, 4200-150 Porto, Portugal
* Correspondence: famaro@ff.up.pt (F.A.); jipinto@ff.up.pt (J.P.); Tel.: +351-220428796 (F.A. & J.P.)

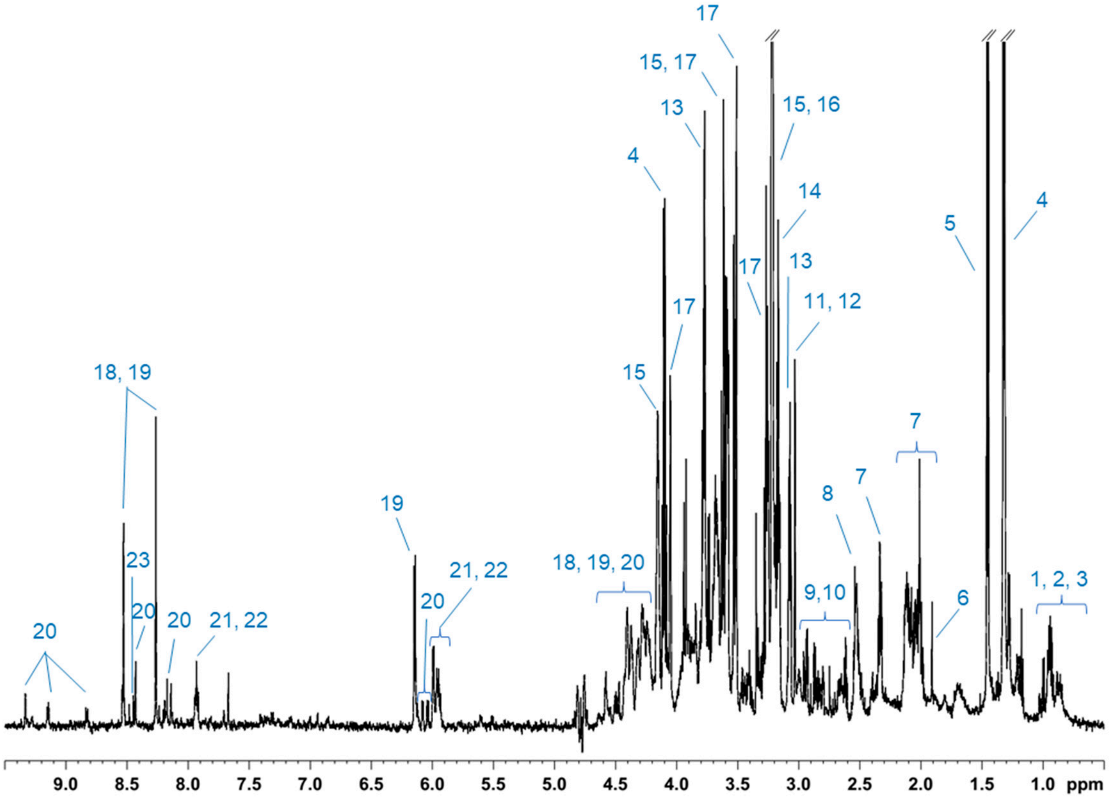

Figure S1. Representative ^{1}H NMR spectrum obtained for the intracellular extracts (endometabolome) of non-exposed (control) Caki-1 cells. 1: isoleucine; 2: leucine; 3: valine; 4: lactate; 5: alanine; 6: acetate; 7: glutamate; 8: glutathione; 9: aspartate; 10: asparagine; 11: creatine; 12: phosphocreatine; 13: ethanolamine; 14: choline; 15: *o*-phosphocholine; 16: glycerophosphocholine; 17: *myo*-inositol; 18: ADP; 19: ATP; 20: NAD+; 21: UDP-glucose; 22: UDP-galactose; 23: formate.

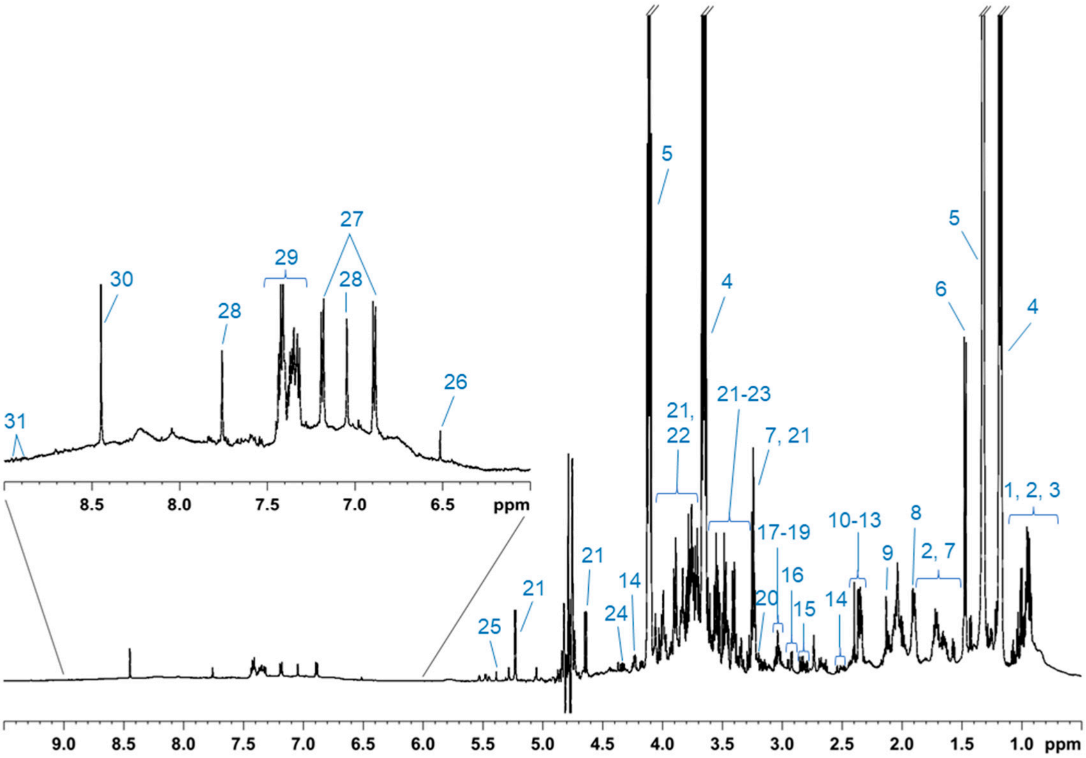

Figure S2. Representative ^{1}H NMR spectrum obtained for the extracellular culture medium (exometabolome) of non-exposed (control) Caki-1 cells. 1: isoleucine; 2: leucine; 3: valine; 4: ethanol; 5: lactate; 6: alanine; 7: arginine; 8: acetate; 9: methionine; 10: glutamate; 11: pyruvate; 12: succinate; 13: glutamine; 14: pyroglutamate; 15: aspartate; 16: asparagine; 17: lysine; 18: creatine; 19: phosphocreatine; 20: choline; 21: glucose; 22: *myo*-inositol; 23: glycine; 24: threonine; 25: glycogen; 26: fumarate; 27: tyrosine; 28: 1-methylhistidine; 29: phenylalanine; 30: formate; 31: 1-methylnicotinamide.

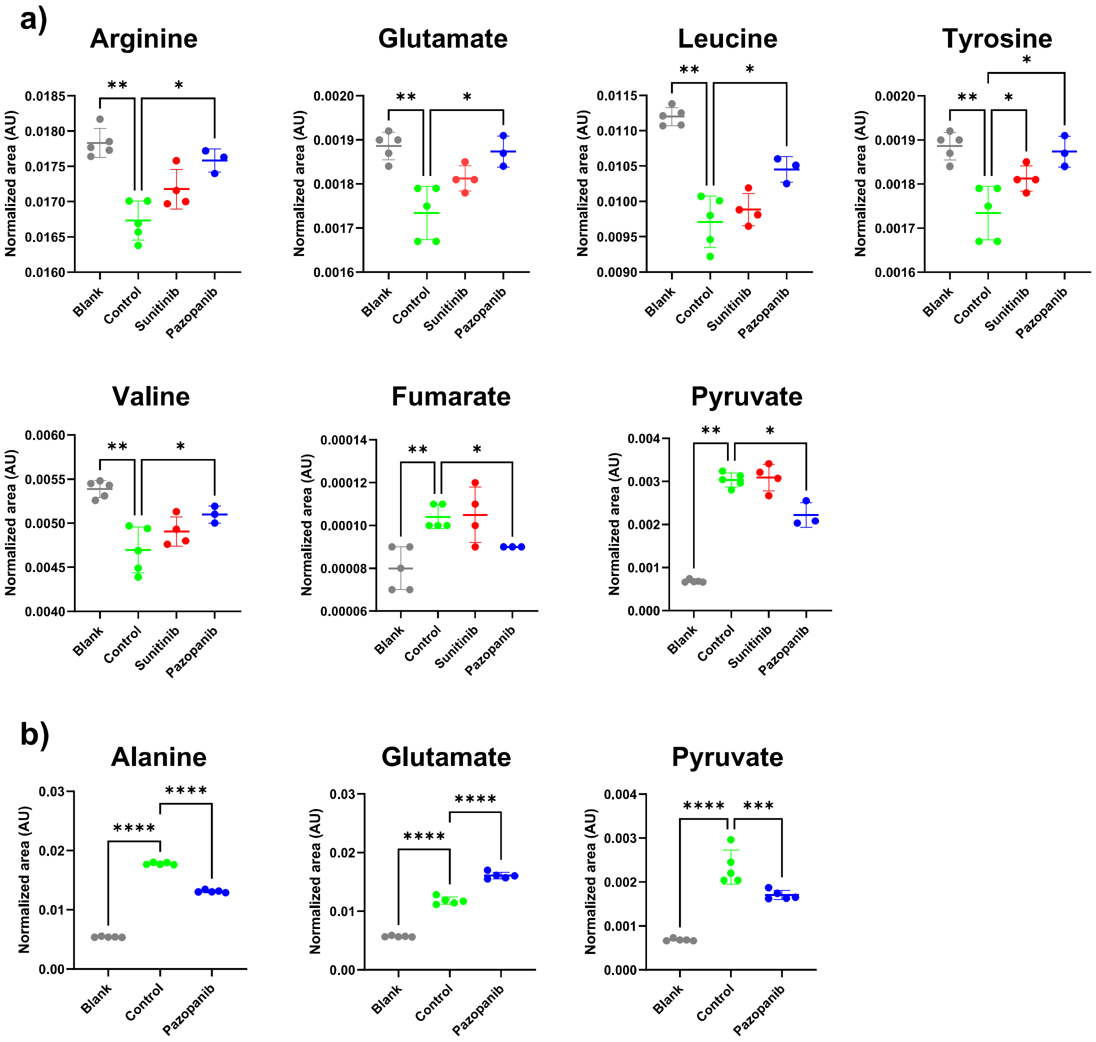

Figure S3. Boxplots of metabolites found significantly altered in the exometabolome (extracellular metabolites present in culture medium). a, b) Boxplots of metabolites altered in Caki-1 and HK-2 cells, respectively. It is possible to observe the differences in terms of consume and excretion compared with blanks (culture medium without cells). The statistical significance was assessed by comparison with the control cells (* p-value < 0.05, ** p-value < 0.01, *** p-value < 0.001, **** p-value < 0.0001).

Table S1. Metabolites identified in the intracellular extracts (endometabolome) of Caki-1 and HK-2 cells using ^{1}H NMR.

No.	Compound	δ ^{1}H in ppm (Multiplicity, Assignment)
1	Isoleucine	0.93 (t); 1.00 (d); 1.25 (m); 1.46 (m); 1.97 (m); 3.66 (d)
2	Leucine	0.94 (d); 0.95 (d); 1.70 (m); 1.69 (m); 3.73 (d)
3	Valine	0.98 (d); 1.03 (d); 2.26 (m); 3.59 (d)
4	Lactate	1.31 (d); 4.09 (q)
5	Alanine	1.47 (d); 3.77 (q)
6	Acetate	1.90 (s)
7	Glutamate	2.04 (m); 2.11 (m); 2.34 (m); 3.74 (q)
8	Glutathione	2.15 (m); 2.54 (m); 2.94 (dd); 2.97 (dd); 3.76 (m); 4.55 (m)
9	Aspartate	2.67 (dd); 2.80 (dd); 3.88 (dd)
10	Asparagine	2.84 (dd); 2.94 (dd); 3.98 (q)
11	Creatine	3.02 (s); 3.91 (s)
12	Phosphocreatine	3.03 (s); 3.93 (s)
13	Ethanolamine	3.13 (t); 3.81 (t)
14	Choline	3.19 (s); 3.50 (t); 4.05 (m)
15	*o*-Phosphocholine	3.21 (s); 3.58 (m); 4.15 (m)
16	Glycerophosphocholine	3.24 (s); 3.68 (m); 3.70 (m); 4.33 (m)
17	*Myo*-inositol	3.26 (t); 3.52 (dd); 3.61 (t); 4.05 (t)
18	ADP	4.22 (m); 4.38 (m); 4.61 (m); 6.15 (d); 8.28 (s); 8.54 (s)
19	ATP	4.22 (m); 4.29 (m); 4.41 (m); 4.62 (m); 6.15 (d); 8.28 (s); 8.55 (s)
20	NAD+	4.23 (m); 4.36 (m); 4,39 (m); 4.42 (m); 4.50 (m); 4.54 (m); 6.04 (d); 6.10 (d); 8.18 (s); 8.19 (t); 8.43 (s); 8.83 (d); 9.14 (d); 9.34 (s)
21	UDP-Glucose	3.49 (t); 3.54 (m); 3.74 (t); 3.76 (dd); 3.84 (d); 4.19 (m); 4.23 (m); 4.27 (s); 4.36 (m); 5.97 (d); 5.98 (d); 7.93 (dd)
22	UDP-Galactose	3.70 (dd); 3.74 (d); 3.90 (dd); 4.02 (dd); 4.16 (m); 4.22 (m); 4.29 (d); 5.97 (d); 7.99 (d)
23	Formate	8.44 (s)

s: singlet, d: doublet, t: triplet, dd: doublet of doublets, m: multiplet.

Table S2. List of metabolites significantly altered between the endometabolome of Caki-1 and HK-2 cells.

Class/Metabolite	Caki-1 *vs.* HK-2		Dysregulated Metabolic Pathways
	ES ± SE	*p*-Value	
Amino acids and derivatives			
Alanine	-5.58 ± 2.83	<0.0001	Aminoacyl-tRNA biosynthesis
Creatine and/or phosphocreatine	-3.80 ± 2.11	0.0159	Arginine and proline metabolism
Glutamate	19.3 ± 9.01	<0.0001	Aminoacyl-tRNA biosynthesis, glutathione metabolism
Phosphocholines			
o-Phosphocholine	-4.43 ± 2.36	0.0001	Glycerophospholipid metabolism
Sugar derivatives			
Myo-inositol	-2.59 ± 1.67	0.0034	Galactose metabolism, phosphatidylinositol signaling system, inositol phosphate metabolism

ES: effect size, SE: standard error. Statistically significance assessed using *t*-test or Mann-Whitney test.

Table S3. Metabolites identified in the extracellular culture medium (exometabolome) of Caki-1 and HK-2 cells using ^{1}H NMR.

No.	Compound	δ ^{1}H in ppm (Multiplicity, Assignment)
1	Isoleucine	0.93 (t); 1.00 (d); 1.25 (m); 1.46 (m); 1.97 (m); 3.66 (d)
2	Leucine	0.94 (d); 0.95 (d); 1.70 (m); 1.69 (m); 3.73 (d)
3	Valine	0.98 (d); 1.03 (d); 2.26 (m); 3.59 (d)
4	Ethanol	1.19 (t); 3.67 (t)
5	Lactate	1.31 (d); 4.09 (q)
6	Alanine	1.47 (d); 3.77 (q)
7	Arginine	1.64 (m); 1.72 (m); 1.89 (m); 1.91 (m); 3.35 (t); 3.76 (t)
8	Acetate	1.90 (s)
9	Methionine	2.11(m); 2.12 (s), 2.18 (m); 2.63 (t); 3.84 (t)
10	Glutamate	2.04 (m); 2.11 (m); 2.34 (m); 3.74 (q)
11	Pyruvate	2.35 (s)
12	Succinate	2.38 (s)
13	Glutamine	2.13 (m); 2.44 (m); 3.76 (t)
14	Pyroglutamate	2.02 (m); 2.39 (t), 2.49 (m), 4.16 (q)
15	Aspartate	2.67 (dd); 2.80 (dd); 3.88 (dd)
16	Asparagine	2.84 (dd); 2.94 (dd); 3.98 (q)
17	Lysine	1.46 (m); 1.72 (m) 1.89 (m); 1.92 (m); 3.01 (t); 3.74 (t)
18	Creatine	3.02 (s); 3.91 (s)
19	Phosphocreatine	3.03 (s); 3.93 (s)
20	Choline	3.19 (s); 3.50 (t); 4.05 (m)
21	Glucose	3.54 (dd); 3.70 (m); 3.72 (m); 3.76 (q); 3.84 (m); 3.85 (dd); 3.91 (dd); 4.65 (d); 5.24 (d)<br>3.23 (t); 3.39 (m); 3.45 (m); 3.50 (dd); 3.71 (m); 3.81(m); 3.88 (dd); 4.63 (d)
22	Myo-inositol	3.26 (t); 3.52 (dd); 3.61 (t); 4.05 (t)
23	Glycine	3.55 (s)
24	Threonine	1.31 (d); 3.57 (d); 4.24 (m)
25	Glycogen	3.40 (m); 3.60 (m); 3.80 (m); 3.96 (s); 5.50 (s)
26	Fumarate	6.50 (s)
27	Tyrosine	3.05 (q); 3.18 (dd); 3.92 (m); 6.89 (d); 7.18 (d)
28	1-Methylhistidine	3.06 (q); 3.17 (dd); 3.68 (s); 3.96 (q); 7.05 (s); 7.76 (s)
29	Phenylalanine	3.12 (m); 3.27 (dd); 3.98 (m); 7.32 (d); 7.36 (m); 7.41 (m)
30	Formate	8.44 (s)
31	1-Methylnicotinamide	4.47 (s); 8.17 (t); 8.89 (d); 8.96 (d); 9.27 (s)

s: singlet, d: doublet, t: triplet, dd: doublet of doublets, m: multiplet.

Table S4. List of metabolites significantly altered between the exometabolome of Caki-1 and HK-2 cells.

Class/Metabolite	Caki-1 *vs.* HK-2<br>ES ± SE	 *p*-Value	Dysregulated Metabolic Pathways
Amino acids			
Arginine	3.88 ± 2.04	0.0003	Aminoacyl-tRNA biosynthesis
Glutamine	3.99 ± 2.08	0.0002	Aminoacyl-tRNA biosynthesis
Glutamate	-6.01 ± 2.86	<0.0001	Aminoacyl-tRNA biosynthesis, glutathione metabolism
Acids			
Lactate	-2.46 ± 1.56	0.0048	Energy metabolism
Succinate	4.08 ± 2.11	0.0002	Energy metabolism
Formate	-4.58 ± 2.30	<0.0001	Energy metabolism
Phosphocholines			
o-Phosphocholine	2.92 ± 1.70	0.0018	Glycerophospholipid metabolism
Sugars and derivatives			
Glucose	2.18 ± 1.47	0.0092	Energy metabolism
Myo-inositol	-1.98 ± 1.42	0.0147	Galactose metabolism, phosphatidylinositol signaling system, inositol phosphate metabolism

ES: effect size, SE: standard error. Statistically significance assessed using *t*-test or Mann-Whitney test.
